# Reflective Distributed Denial of Service Detection: A Novel Model Utilizing Binary Particle Swarm Optimization—Simulated Annealing for Feature Selection and Gray Wolf Optimization-Optimized LightGBM Algorithm

**DOI:** 10.3390/s24196179

**Published:** 2024-09-24

**Authors:** Daoqi Han, Honghui Li, Xueliang Fu

**Affiliations:** College of Computer and Information Engineering, Inner Mongolia Agricultural University, Hohhot 010018, China; 15623587331@163.com (D.H.); fuxl_imau@163.com (X.F.)

**Keywords:** intrusion detection system (IDS), feature selection (FS), binary particle swarm optimization (BPSO), simulated annealing (SA), gray wolf optimization (GWO), LightGBM

## Abstract

The fast growth of the Internet has made network security problems more noticeable, so intrusion detection systems (IDSs) have become a crucial tool for maintaining network security. IDSs guarantee the normal operation of the network by tracking network traffic and spotting possible assaults, thereby safeguarding data security. However, traditional intrusion detection methods encounter several issues such as low detection efficiency and prolonged detection time when dealing with massive and high-dimensional data. Therefore, feature selection (FS) is particularly important in IDSs. By selecting the most representative features, it can not only improve the detection accuracy but also significantly reduce the computational complexity and attack detection time. This work proposes a new FS approach, BPSO-SA, that is based on the Binary Particle Swarm Optimization (BPSO) and Simulated Annealing (SA) algorithms. It combines these with the Gray Wolf Optimization (GWO) algorithm to optimize the LightGBM model, thereby building a new type of reflective Distributed Denial of Service (DDoS) attack detection model. The BPSO-SA algorithm enhances the global search capability of Particle Swarm Optimization (PSO) using the SA mechanism and effectively screens out the optimal feature subset; the GWO algorithm optimizes the hyperparameters of LightGBM by simulating the group hunting behavior of gray wolves to enhance the detection performance of the model. While showing great resilience and generalizing power, the experimental results show that the proposed reflective DDoS attack detection model surpasses conventional methods in terms of detection accuracy, precision, recall, F1-score, and prediction time.

## 1. Introduction

Given the Internet’s quick development, global information exchange and business activities are increasingly dependent on network communications. Network security issues are becoming increasingly important. Distributed Denial of Service (DDoS) attacks, known for their destructive nature, are escalating in terms of both frequency and complexity, posing significant threats to network infrastructure and information security. In the first quarter of 2024, the StormWall report [1] showed that the average number of devices in botnets increased five-fold, from 4000 to 20,000, enough to threaten global Internet stability. DDoS attacks against government departments accounted for 34%, a year-on-year increase of 47%. In addition, the proportion of hacker-driven attacks relative to profit-making DDoS attacks is also steadily increasing. In order to effectively respond to these threats, intrusion detection systems (IDSs) have become a key tool for protecting network security [2,3,4]. IDSs ensure network stability and data security by continuously monitoring network traffic and identifying and thwarting potential attack behaviors.

When handling extensive and complex datasets, traditional intrusion detection algorithms frequently encounter issues such as low detection efficiency [5,6] and long detection times [7]. Feature selection (FS) has emerged as a key link in IDSs for improving detection accuracy and efficiency [8]. By identifying the most representative features, FS not only boosts detection performance but also significantly reduces computational complexity and detection time. Machine learning models have substantial advantages over deep learning models in FS and DDoS attack detection [9]. Although deep learning models perform well in processing complex data, their shortcomings of high resource consumption and poor real-time performance [10] limit their application in real-time intrusion detection.

In this context, integrating robust FS methods with effective classification techniques can dramatically improve IDS efficiency [11]. One of the methods to implement FS is based on the meta-heuristic algorithm [12]. Metaheuristic algorithms may effectively find optimal feature subsets in complex feature spaces by simulating the natural optimization process, which improves the model’s detection performance and efficiency [13]. These algorithms perform well when dealing with high-dimensional data, balancing exploration and exploitation and avoiding falling into local optimal solutions.

Current intrusion detection research mostly focuses on the binary classification problem [14], which involves discriminating between regular and attack traffic. However, the multi-classification problem is significant in practical applications, particularly for detecting various types of DDoS attacks. A major challenge facing multi-classification problems is that the detection accuracy of minority categories is low, while the accuracy of mainstream categories is often higher [15]. An imbalance like this affects the overall effectiveness of the detection system. In addition, reflective DDoS attacks are more difficult to detect than amplified DDoS attacks due to their concealment and complexity, and effective detection methods are urgently needed to deal with them.

Facing the above challenges, this study introduces a novel FS method that combines Binary Particle Swarm Optimization (BPSO) and Simulated Annealing (SA) algorithms, alongside the innovative Gray Wolf Optimization (GWO) algorithm, to optimize the LightGBM model specifically for detecting reflective DDoS attacks. Reflective DDoS attacks, characterized by their concealment and complexity, present significant challenges in detection compared to amplified DDoS attacks, highlighting the urgency for effective detection methods.

Our approach not only tackles the multi-classification problem, which is crucial for identifying diverse DDoS attack types, but it also addresses the challenge of low detection accuracy for minority categories [15]. The proposed BPSO-SA FS technique facilitates the extraction of a more efficient feature subset, thus enhancing the model’s performance in real-time environments. Additionally, GWO optimizes the hyperparameters of LightGBM, thereby improving multi-classification detection capabilities and accuracy for reflective DDoS attacks.

The key contributions of this study are outlined as follows:

1. A novel FS method, BPSO-SA, employs the SA algorithm to optimize BPSO, resulting in a smaller and more efficient feature subset that reduces attack detection time.
2. The application of GWO for tuning LightGBM hyperparameters enhances the multi-classification detection of network intrusions, particularly improving detection accuracy for reflective DDoS attacks.
3. A comparative evaluation of the BPSO-SA FS approach against four other FS methods, alongside an assessment of the enhanced LightGBM detection model against six other machine learning models.

The remainder of this paper is organized as follows: Section 2 discusses IDS-related work. Section 3 elaborates on the proposed IDS attack detection model and FS mechanism. The experimental design and data analysis are provided in Section 4. Section 5 summarizes the research presented in this publication.

## 2. Related Work

Particularly in DDoS detection, IDSs have drawn growing interest from researchers over the past few years as a research hotspot in the field of network security. Given that network traffic data are often large-scale and high-dimensional, it is essential to employ various techniques and methods for dimensionality reduction. This step is crucial for advancing the operational efficiency of an Intrusion Detection System (IDS). While network attacks can be identified using deep learning algorithms, the training procedure typically takes a huge quantity of data and processing resources.

Nguyen et al. [16] developed a DDoS detection strategy that incorporates deep learning and a Gaussian mixture model (GMM). Specifically, they integrated a BI-LSTM with the GMM, achieving 94% in terms of recall, precision, and accuracy. However, this method requires manual intervention to identify and label unknown traffic captured by the GMM, which limits its automation ability and universality.

Javeed et al. [17] engineered a solution for identifying emerging network threats within the Internet of Things environment through the integration of software-defined networking (SDN) architecture and a hybrid deep learning algorithm. The framework shows a low false-positive rate and good accuracy.

Xu et al. [18] developed an improved bidirectional generative adversarial network (Bi-GAN) model, which reduced training overhead and improved detection performance by improving the training strategy.

Aldhyani et al. [6] suggested a CNN-LSTM-based deep learning model for detecting DDoS assaults in the Agriculture 4.0 environment, and it performed well. However, this study is constrained by conventional datasets, and its efficiency in real-world contexts requires further testing.

The combination of the deep learning intrusion detection model (HD-IDM) introduced by Ahmad et al. [19] combines GRU and LSTM classifiers. It improves the accuracy of detecting complex network data patterns through weighted average fusion, achieving an accuracy of 99.91%. However, this model has a high dependence on labeled data and has challenges in processing unlabeled data and adapting to new threats.

On the CICDDoS2019 and InSDN datasets, Setitra et al. [14] optimized the MLP-CNN model and yielded 99.95% and 99.98% accuracy, respectively.

Chen et al. [20] proposed an adversarial DBN-LSTM method to improve the system’s robustness against hostile assaults by generating adversarial datasets.

Wang et al. [21] proposed the DDosTC model, integrating scalable and efficient Transformer and CNN architectures for DDoS attack detection in SDN. The model outperforms existing methods in multiple evaluation indicators. However, it faces challenges commonly faced by deep learning models, such as dependence on a large amount of labeled data and possible overfitting risks.

Although deep learning has demonstrated excellent performance and potential in DDoS attack detection, it still faces some challenges. These include insufficient model generalization ability and a large number of deep learning model parameters that consume hardware resources. To overcome these challenges, researchers have gradually turned their attention to the combination of FS and traditional machine learning techniques. FS technology can effectively reduce data dimensionality, improving the training efficiency and generalization ability of the model. As a result, it plays an important role in intrusion detection systems (IDSs) and network security.

Arden et al. [22] conducted a detailed comparison of hyperparameter tuning methods for different machine learning algorithms by evaluating the combination of six algorithms and six hyperparameter tuning methods (random search, grid search, Bayesian optimization, genetic algorithm, SHERPA, and Optuna). The findings revealed that in some cases, random forest performed best even without hyperparameter tuning. This provides researchers with important insights into the applicability and efficiency of hyperparameter tuning in different algorithms.

Hsu et al. [23] suggested a real-time DDoS detection system based on random forest, GBDT, XGBoost, LightGBM, CatBoost, K-NearestNeighbor (KNN), and MLP by evaluating the performance of machine learning models in a real network environment. By using real campus network traffic log data for evaluation, the study showed the applicability and effectiveness of the method in real time on a large-scale network, further verifying the potential of machine learning in practical applications.

Furthermore, in the realm of automotive network security, Altalbe [24] suggested an IDS (FFS-IDS). The system effectively detects Denial of Service (DoS), gear deception, and RPM deception attacks in car networks by combining several characteristics collected from raw network traffic and employing ensemble learning techniques for categorization. This study demonstrates the advantages of FS and ensemble learning in improving detection accuracy and real-time performance.

Tu et al. [8] introduced an enhanced RFECV algorithm, termed ImprovedRFECV, which strengthens the robustness and generalization of the optimal feature subset. This is achieved through the integration of random sampling, L1 and L2 regularization, and a multi-model ensemble framework. Their research demonstrates that ImprovedRFECV excels across various datasets, significantly boosting FS efficiency and model accuracy.

In terms of attack detection in the IoT environment, Hasan et al. [25] examined the performance of numerous machine learning models. The results show that decision tree (DT), random forest (RF), and ANN perform best in detecting attacks and anomalies in IoT sensors, providing strong support for building intelligent, secure, and reliable IoT infrastructure.

Using the most recent CICDDoS2019 dataset, Alzahrani [26] examined six different machine learning algorithms (KNN, Support Vector Machine (SVM), NB, DT, RF, and Logistic Regression (LR)) for DDoS attack detection. The outcomes demonstrate that DT and RF offer an effective solution for DDoS attack detection, performing well in terms of accuracy.

These research works demonstrate the great potential of combining FS with machine learning in IDSs and network security. By deeply analyzing the performance of different algorithms and tuning methods, these studies provide important references and guidance for solving the problem of DDoS attack detection. In Table 1, we compare the approaches, advantages, and drawbacks of related works.

## 3. Proposed DDoS Attack Detection Model

**Figure 1:**
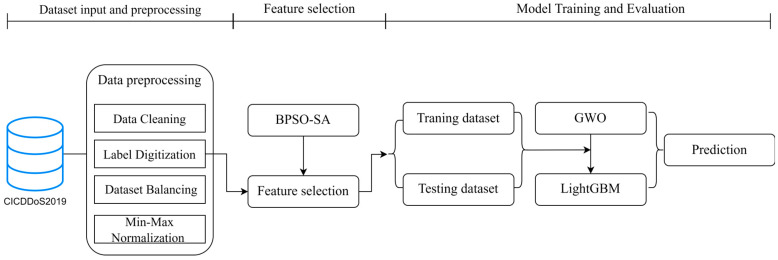
DDoS attack detection architecture diagram.

This section presents the newly proposed DDoS attack detection model. The model’s architecture is displayed in Figure 1. The dataset input and preprocessing part, the FS part, and the model training and assessment part are its three components.

### 3.1. Dataset Input and Preprocessing

This section describes the selected dataset and the data preprocessing process.

#### 3.1.1. Dataset

The CICDDoS2019 [27] dataset, compiled by the Canadian Institute for Cybersecurity (CIC), is the most thorough and recent DDoS attack dataset currently accessible. The dataset contains 88 network flow features, including detailed information such as protocols and target ports, and covers a wealth of DDoS attack behavior records.

This dataset’s attack types are classified into two categories: reflection-based and exploit-based attacks. Reflection-based DDoS attacks take advantage of legitimate servers. Exploitation-based DDoS attacks exploit flaws in TCP and UDP connection protocols.

#### 3.1.2. Dataset Preprocessing

In this study, we performed several preprocessing steps on the CICDDoS2019 dataset to ensure the quality and effectiveness of the data used for training the model. Initially, we removed samples containing infinite values and null values to maintain the integrity of the dataset. Furthermore, we eliminated eight features deemed irrelevant for training purposes, specifically Flow ID, Source IP, Destination IP, Source Port, Destination Port, Timestamp, Fwd Header Length.1, and SimilarHTTP. Additionally, we excluded twelve features that contained all zero values, which included Bwd PSH Flags, Fwd URG Flags, Bwd URG Flags, FIN Flag Cnt, PSH Flag Cnt, ECE Flag Cnt, Fwd Byts/b Avg, Fwd Pkts/b Avg, Fwd Blk Rate Avg, Bwd Byts/b Avg, Bwd Pkts/b Avg, and Bwd Blk Rate Avg. Table 2 shows the 68 features contained in the CICDDoS2019 dataset after the above processing and their corresponding serial numbers.

To focus on reflective DDoS attacks, we extracted 100,000 samples of various reflective DDoS attacks from the 12 January 2019 CSV file in the dataset. Given the limited availability of normal traffic samples, we applied the SMOTE [28] method to generate additional normal traffic samples, thus balancing the dataset effectively. Each sample was then numerically labeled in accordance with the specifications of the machine learning algorithm. To ensure all features were on a comparable scale, we applied Min–Max normalization [29], which transformed all sample values to a range between 0 and 1. A summary of the dataset after these preprocessing steps is provided in Table 3.

### 3.2. Proposed BPSO-SA FS Method

This section mainly introduces the BPSO algorithm, SA algorithm, and the proposed BPSO-SA FS algorithm used in this paper.

#### 3.2.1. BPSO

The PSO algorithm [30] is motivated by bird feeding behavior and optimized by modeling the movement of many particles. In FS, the PSO algorithm treats the feature space as a search space and each particle represents a subset of features. The particles iteratively navigate the search space, continually updating their positions based on their individual best solution and the population’s global best solution. This approach allows the particles to explore efficiently over the feature subset.

The particles consider both their own experience and the global optimal solution during the update process, which ensures the comprehensiveness and accuracy of FS. This method enhances the model’s accuracy and generalization while also greatly optimizing its efficiency by removing unnecessary characteristics while maintaining critical ones.

Suppose N particles form a colony in a D-dimensional target search space. With the i particle, $X_{i}$ is represented as a D-dimensional vector:

(1) $$X_{i}=\left(x_{i1},x_{i2},\cdot \cdot \cdot x_{iD}\right),i=1,2,\cdot \cdot \cdot ,N$$

The flying speed of the i particle is also a D-dimensional, recorded as follows:

(2) $$V_{i}=\left(v_{i1},v_{i2},\cdot \cdot \cdot ,v_{iD}\right),i=1,2,\cdot \cdot \cdot ,N$$

When the i particle of the t generation evolves to the t+1 generation, it is updated according to the following Equations (3) and (4):

(3) $$v_{ij}\left(t+1\right)={wv}_{ij}\left(t\right)+c_{1}r_{1}\left(t\right)\left[p_{ij}\left(t\right)-x_{ij}\left(t\right)\right]+c_{2}r_{2}\left(t\right)\left[p_{gj}\left(t\right)-x_{ij}\left(t\right)\right]$$

(4) $$x_{ij}\left(t+1\right)=x_{ij}\left(t\right)+v_{ij}\left(t+1\right)$$

Among them, $v_{ij}\left(t+1\right)$ represents the velocity of the i-th particle in the j-th dimension at the t+1 iteration; w is the inertia weight; $v_{ij}(t)$ represents the ith particle in the j-th dimensional at the t iteration; $c_{1}$ and $c_{2}$ represent the individual learning coefficient and the global learning coefficient, respectively, which are used to change the maximum step size of the individual and group ideal positions; $r_{1}$ and $r_{2}$ are random values spread between [0, 1], known as inertia factors, and the bigger the value, the wider the search range; and $p_{ij}(t)$ and $p_{gj}(t)$ represent the position of the local optimal solution and the position of the global optimal solution discovered by the entire particle group in the j-th dimension at the t iteration, respectively.

The main purpose of the conventional PSO method is to resolve issues involving continuous variables. Since the FS problem is a typical combinatorial optimization problem in discrete space, the standard PSO algorithm cannot be directly applied. Based on this, Kennedy and Eberhart proposed the BPSO algorithm in 1997 [31], whose particle position component has only two states: 0 and 1. For BPSO, all states of the position space can be regarded as a hypercube. A single particle represents a vertex of the hypercube, and the search process causes the particle to move between the vertices of the hypercube. Although the particle position component in BPSO has only two values, 0 and 1, the velocity update Formula (3) is still applicable, and the calculation result is still a real number. Another difference between BPSO and standard PSO is that BPSO abandons the position update Formula (4) and uses the sigmoid conversion function to convert the obtained real velocity component value to the interval [0, 1]. The converted value is then compared to a randomly generated value. If the random number is smaller than the converted number, the position component is set to 1; otherwise, the position component is set to 0. The position conversion process can be formalized as follows:

(5) $$T\left(v_{ij}\left(t+1\right)\right)=\frac{1}{1+e-v_{ij}\left(t\right)}$$

(6) $$x_{ij}\left(t+1\right)=\left\{\begin{array}{c} 0,rand<T\left(v_{ij}\left(t+1\right)\right)\\ 1,rand\geq T\left(v_{ij}\left(t+1\right)\right) \end{array}\right.$$

Among them, e represents the base of natural logarithms, and rand represents a random number.

#### 3.2.2. SA

The SA algorithm draws on the principle of solid annealing. It optimizes by simulating the disordered motion of particles at high temperatures and the process of gradually tending to order as the temperature decreases. In FS, the SA algorithm starts at a high temperature and allows a larger range of feature subsets to change in order to explore multiple areas of the feature space. As the temperature gradually decreases, the algorithm gradually converges to a better feature subset and eventually reaches the optimal solution for FS. This method can gradually find the feature combination that can best improve model performance while avoiding falling into a local optimum.

The temperature update formula is as follows:

(7) $$T=\max\left(\alpha \times T,T_{min}\right)$$

Among them, T is the present temperature, α is the temperature reduction coefficient, and $T_{min}$ is the minimum temperature.

One of the crucial parameters of the SA algorithm is the probability of accepting a new solution. A reasonable acceptance probability can help the algorithm strike a balance between global search and local search, thereby improving the performance of the algorithm [32]. According to the fitness difference and the current temperature, the smaller the temperature and the greater the fitness difference, the smaller the acceptance probability. The acceptance probability calculation formula is as follows:

(8) $$acceptance\_prob=e^{-\frac{delta\_fitness}{T}}$$

Among them, delta_fitness is the difference between the present fitness and the best fitness.

#### 3.2.3. BPSO-SA

**Figure 2:**
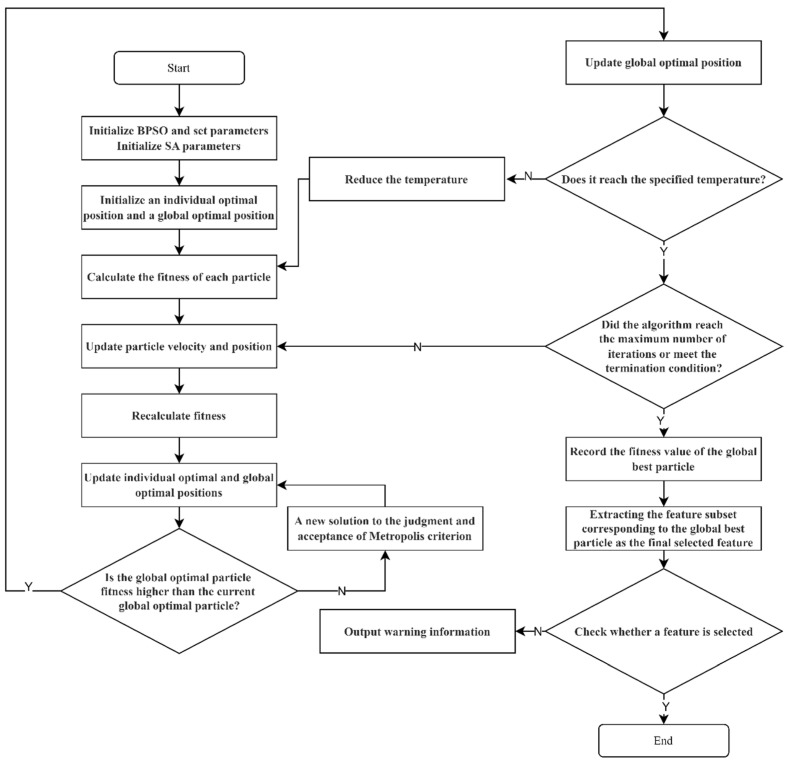
BPSO-SA algorithm flow chart.

Figure 2 depicts the process of the BPSO-SA algorithm. In the improved BPSO algorithm, we combined the core ideas of the SA algorithm and improved the Particle Swarm Optimization process mainly in the following aspects:

1. Introduction of temperature control mechanism: In the conventional BPSO algorithm, particle position updates typically depend on individual and global best solutions, without considering the possibility of accepting inferior solutions. In our improved algorithm, we introduce a temperature control mechanism inspired by SA, such as Formula (7). At the early stages, higher temperatures allow for the particles to accept suboptimal solutions, promoting broader exploration of the solution space. As the temperature gradually decreases, particles become more conservative, increasingly relying on known high-quality solutions, thereby reducing the probability of accepting poor solutions. This approach helps the algorithm better navigate toward a global optimal solution.
2. Incorporation of fitness difference: The update of particle positions in BPSO-SA is influenced by SA through an acceptance criterion. When a particle is updated to a new position, SA determines whether this new position is accepted based on a probability related to the current temperature and the difference in fitness values between the new and old positions. This probability can be expressed as Formula (9). Specifically, the fitness difference is defined as the difference between the current fitness of the particle swarm and the individual best fitness. A greater fitness difference raises the likelihood of accepting a suboptimal solution, which helps prevent particles from becoming stuck in local optima. As the SA process progresses and temperature drops, the impact of the fitness difference on particles lessens, causing the algorithm to increasingly emphasize local optimization in the later stages of the search.

(9) $$acceptance_{prob}=e^{-\frac{\left({accuracy}_{new}-{accuracy}_{current}\right)}{T}}$$

Our previous work [33] proved the effectiveness of DT in DDoS attack detection, so DT is used as the evaluator of the BPSO-SA fitness function.

Figure 2 depicts the proposed BPSO-SA algorithm, which consists of the following steps:

Step 1: Load the dataset and initialize the particle group by setting the number of particles and features. Each particle is randomly assigned a feature selection value (0 or 1), indicating whether the feature is selected.

Step 2: Initialize the velocity of each particle to zero, indicating that the change in the feature selection is zero at the beginning.

Step 3: Evaluate the fitness of each particle, which reflects the quality of the feature selection by considering both the classifier’s accuracy and the number of selected features.

Step 4: Initialize both the individual and the global best positions.

Step 5: Define the number of iterations along with the inertia weight, individual learning factor, and group learning factor. Update the particle’s position and velocity, recalculate fitness, and refresh the individual and global best positions. Integrate the Simulated Annealing algorithm to fine-tune the individual best position while progressively lowering the temperature.

Step 6: Extract the selected feature index from the global best particle. If no feature is selected, use all features.

Step 7: Use the DT classifier to predict the test set and then calculate and report the classifier’s accuracy on that set.

Step 8: Select the final features by evaluating the quantity and accuracy of the chosen features.

The proposed BPSO-SA algorithm is represented by the following pseudo code (Algorithm 1):
**Algorithm 1.** BPSO-SA pseudo code1: input: Preprocessed Data (CICDDoS2019 Dataset) 2: output: Selected feature subset 3: initialize population of particles and velocities 4: initialize the *T*, *α*, and *Tmin* 5: while t < maximum number of iterations: 6: for each particle: 7:   calculate the velocity of particle by Equation (3) 8:   calculate the fitness of all particles 9:   updating position and fitness of particles 10:  if new position better than current: 11:    accept new position 12:    chose the particle of best fitness value and the Gbest of all particles 13:    else: 14:     calculate the acceptance probability of particle by Equation (8) 15:     if rand() < acceptance_probability: 16:     accept new position 17:     update particle position by Equations (5) and (6) 18: end for 19: reduce the temperature 20: End while

### 3.3. Model Training and Evaluation

Traditional machine learning models have several advantages over deep learning models, including minimal computational resource needs, short training time, fast detection speed, and ease of model interpretation [22,23]. These characteristics enable them to perform well in application scenarios with limited hardware resources that require a fast response and real-time feedback. Therefore, this article selects seven machine learning models for comparative analysis.

#### 3.3.1. Split Dataset

This study uses the train_test_split function from the Sklearn library to divide the dataset in a 7:3 ratio, with 70% allocated for training and 30% for testing, to ensure the model’s predictive accuracy.

#### 3.3.2. Model Selection

In order to achieve the efficient classification of datasets, this paper selects and compares multiple machine learning models. These models include DT, RF, NB, KNN, LR, LightGBM, and XGBoost. The reason for choosing these models is that they perform well in different application scenarios and datasets and have different algorithmic characteristics and advantages.

DT: DT is a model that performs classification or regression by recursively splitting data into smaller subsets. The basic principle is to start from the root node and select the best split point by comparing the feature values to generate a tree structure. Advantages include easy to understand and explain, no feature standardization, ability to handle numerical and categorical data, and not susceptible to missing values.

RF: RF comprises multiple DTs that enhance model accuracy and stability through ensemble learning. During training, each tree randomly selects samples and features from the dataset and determines the classification result through voting. Its advantages include high accuracy, robust resistance to overfitting, capability to handle complex data, and automatic feature selection.

NB: The classification method relies on Bayes’ theorem and assumes that features are independent of each other. Classification is performed by calculating the posterior probability of each category. Advantages include fast training speed, good performance on small-scale data, ability to handle multi-class problems, and insensitivity to missing data.

KNN: KNN is an instance-based learning algorithm that performs classification or regression by calculating the distance between a new sample and all samples in the training set. The category of a new sample is determined by the categories of its nearest K neighbors. Advantages include simplicity and intuitiveness, no training process, and applicability to classification tasks with a small amount of data.

LR: LR is a widely used classification model that applies a logistic function to convert input features to category probabilities and estimates model parameters by maximizing the likelihood function. Advantages include it being a simple model, that it is easy to implement and explain, its applicability to binary classification problems, and its processing of high-dimensional sparse data.

LightGBM: LightGBM is an efficient framework based on gradient boosted decision tree (GBDT), which uses the histogram algorithm and leaf growth strategy to improve training speed and reduce memory consumption. Advantages include strong ability to handle large-scale data, fast training speed, low memory usage, support for parallelism, and GPU acceleration.

XGBoost: XGBoost is an advanced implementation of GBDT that enhances model accuracy and efficiency by utilizing weighted splitting and pruning strategies. It also supports L1 and L2 regularization to prevent overfitting. Advantages include high prediction performance, strong flexibility, support for parallel and distributed computing, and the ability to handle missing values.

### 3.4. Reflective DDoS Attack Multi-Classification Detection Method Based on GWO of LightGBM Hyperparameters

Mirjalili et al. devised the GWO, a nature-inspired optimization method [34]. The GWO algorithm has significant advantages in optimizing hyperparameters. First, the GWO algorithm simulates the hunting behavior of gray wolves and can effectively explore and utilize the search space to find a better parameter combination [35]. Compared with other optimization algorithms, GWO shows stronger global search capabilities and convergence speed when dealing with high-dimensional complex problems [36]. In addition, the GWO algorithm requires fewer adjustment parameters, is simple to use, and has demonstrated high optimization performance in a variety of applications [37].

Compared to traditional grid search [38] or random search [39] methods, the GWO is capable of quickly identifying parameter configurations that are close to the global optimum, effectively avoiding local optima. While grid search systematically explores the parameter space, its computational complexity increases dramatically in high-dimensional settings, often requiring substantial computational resources and time, particularly when the number of hyperparameters is large. Additionally, the fixed step size in grid search may lead to the insufficient exploration of critical parameter regions, resulting in the potential omission of better solutions. Random search, while more flexible and capable of alleviating some computational burden, introduces a level of randomness that can reduce the likelihood of finding the optimal solution. Furthermore, its lack of systematic coverage of the entire parameter space may lead to unstable results.

In contrast, GWO’s adaptive search mechanism and its simulation of natural hunting behavior enable a better balance between exploration and exploitation, which not only enhances the classification performance of the model but also significantly reduces computational time and resource usage when optimizing the hyperparameters of LightGBM.

The algorithm for optimizing LightGBM hyperparameters using GWO is expressed in the following pseudo code (Algorithm 2):
**Algorithm 2.** GWO pseudo code1: input: Preprocessed Data (CICDDoS2019 Dataset) 2: output Best Hyperparameters 3: set GWO parameters 4: initialize Alpha_pos, Beta_pos, Delta_pos as zero vectors 5: initialize Alpha_score, Beta_score, Delta_score as infinity 6: randomly initialize search agent positions (Positions) 7: initialize convergence curve (Convergence_curve) 8: initialize iterations and accuracy 9: while current iteration < maximum number of iterations: 10:  for each wolf i in Positions: 11:    update alpha, beta, and delta wolves 12:    set LightGBM parameters 13:    calculate fitness 14:    update Alpha, Beta, and Delta positions and scores 15:  update positions 16:  update iterations and accuracy 17: End while

### 3.5. Evaluation Indicators

Accuracy: accuracy measures the proportion of correctly classified instances out of the total number of instances in a dataset.

(10) $$Accuracy=\frac{TP+TN}{TP+TN+FP+FN}\times 100$$

Among them, true positive (TP) refers to attack packets that are correctly identified as attacks, false-positive (FP) denotes benign packets that are mistakenly classified as attacks, true negative (TN) indicates benign packets correctly recognized as normal, and false negative (FN) signifies attack packets that are wrongly categorized as normal. These metrics are derived from the confusion matrix.

Precision: precision represents the ability to identify true positives in all positive predictions.

(11) $$Precision=\frac{TP}{TP+FP}\times 100$$

Recall: recall, also known as sensitivity, measures the proportion of true positive instances correctly identified by a model out of all the actual positive instances.

(12) $$Recall=\frac{TP}{TP+FN}\times 100$$

F1-score: The F1-score is the harmonic mean of precision and recall, providing a single metric that balances both the precision and the recall of a model.

(13) $$F1-score=2\times \frac{Precision\times Recall}{Precision+Recall}\times 100$$

Prediction time: prediction time is the time it takes from input data to the model generating an output prediction.

## 4. Experimental Results and Analysis

This section presents the experimental results of the BPSO-SA FS method and evaluates the performance of seven machine learning models, including LightGBM optimized with GWO.

### 4.1. Experimental Environment

The experiments were conducted on a Windows 10 64-bit operating system with 24 GB of RAM and an Intel Core i5-8250U CPU @ 1.60 GHz. Python version 3.11.5 and Scikit-learn 1.3.1 were utilized for the machine learning tasks. Scikit-learn provides a wealth of tools for data preprocessing, model selection, evaluation, and tuning. It is suitable for various machine learning tasks and is one of the preferred machine learning libraries for data scientists and engineers.

### 4.2. Feature Selection

#### 4.2.1. Use BPSO-SA to Determine the Number of Selected Features

To clarify the distinction between the features picked by BPSO-SA and other FS approaches, the experimental steps are as follows: First, use the BPSO-SA method to select features and determine the number of selected features. Then, use XGBoost, RF, DT, and IG to select features and finally compare the features selected by BPSO-SA, XGBoost, RF, DT, and IG to evaluate their advantages and disadvantages. The initial parameter settings of the BPSO-SA FS method are shown in Table 4.

**Figure 3:**
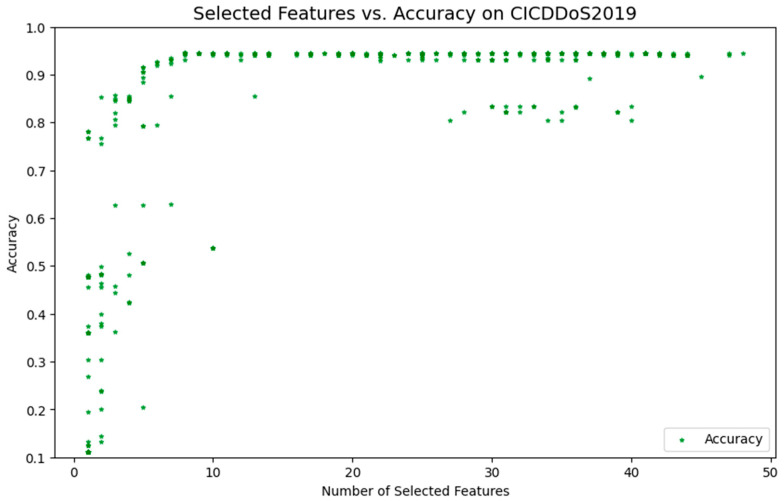
FS using the BPSO-SA method.

Figure 3 illustrates how varying the number of features impacts model accuracy when using the BPSO-SA method. Within a specific range, an increase in the number of selected features generally leads to improved model accuracy. As seen in Figure 3, choosing eight features as the starting point of the model is a wise decision that can balance model performance, complexity, and computational cost. In terms of the importance of FS, although eight features may not yet achieve the highest accuracy, they are sufficient to capture the key information in the data and provide a reasonable starting point. From the accuracy estimation, eight features are in the early stages of accuracy growth, which means there is potential for further optimization. Fewer features minimize model complexity and computer resource needs while also lowering the danger of overfitting and boosting the model’s generalizability. Eight features provide a basis for experimentation. By gradually increasing the number of features, the change in accuracy can be observed, and the optimal number of features can be found. With limited resources, such a choice can also find a balance between cost-effectiveness. In summary, the selection of eight features provides a solid foundation for further optimization of and improvement in the model.

**Figure 4:**
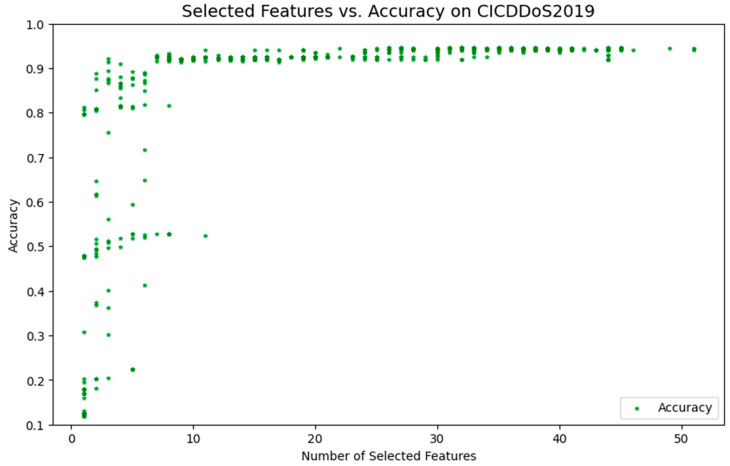
FS using BPSO method.

Similarly, this paper uses the BPSO method without the SA algorithm for FS, and the results are displayed in Figure 4. According to the information in Figure 4, eight features are selected for further optimization and comparison of the model. Similarly, this paper uses the BPSO method without the SA algorithm for FS, and Figure 3 illustrates the results. According to the information in Figure 4, eight features are selected for further optimization and comparison of the model.

Comparing Figure 3 and Figure 4, we can clearly see the significant advantages of the BPSO-SA method. Within the 0–10 feature interval, the points in the BPSO-SA method image converge more smoothly to the global optimal solution with smaller fluctuations. This indicates that BPSO-SA has stronger exploration capabilities in the early stage. The integration of SA into BPSO plays a key role here. Specifically, SA introduces a probabilistic acceptance criterion that allows for the acceptance of suboptimal solutions at the early stages of the optimization process. This mechanism enhances the exploration capability of BPSO, enabling it to escape local optima and explore a wider range of the solution space, especially in the early iterations when the system temperature is higher.

At the same time, when approaching the global optimal solution, BPSO-SA can conduct more detailed searches, thereby improving the stability and accuracy of the optimization results. As the temperature gradually decreases in SA, the algorithm reduces the likelihood of accepting worse solutions, focusing on exploitation around the global optimum, which improves the precision of the final solution.

Within the 25–40 feature interval, the points in the BPSO-SA method image are more widely distributed, demonstrating its stronger exploration ability in the entire solution space. This wide distribution shows that BPSO-SA can effectively avoid falling into the local optimal solution prematurely. The dynamic temperature control mechanism in SA ensures that the balance between exploration and exploitation is maintained throughout the optimization process, allowing BPSO-SA to adapt to complex search spaces and enhance its global search capabilities.

Overall, the BPSO-SA method enhances both global search and local search capabilities by combining the SA mechanism. This combination not only improves the model’s ability to escape local optima through probabilistic jumps but also ensures a more focused and accurate search near the optimal solution as the temperature decreases. This integration allows BPSO-SA to show better performance when dealing with complex feature selection problems, providing superior robustness and adaptability in high-dimensional spaces.

#### 4.2.2. ML-Based FS

Based on the eight features selected by the BPSO-SA method, we also selected eight features using the XGBoost, RF, DT, and IG methods. Table 5 displays the results of the selection process.

Bwd IAT Std (No. 10) can reveal the fluctuation in the arrival time of attack traffic, which is usually large; Flow Byts/s (No. 20) helps detect abnormally high traffic because attacks will significantly increase traffic; and Flow Duration (No. 21) reflects the difference in traffic duration, and attack traffic is often shorter. A large amount of data are generated within. Flow IAT Max (No. 22) displays time interval fluctuations in attack traffic, Fwd Pkt Len Mean (No. 35) and Fwd Seg Size Min (No. 41) can reveal the pattern of attack packet length, and Pkt Len Var (No. 54) captures abnormal changes in packet length in attack traffic. Finally, Unnamed: 0 (No. 67) is usually an index column in the dataset. Taken together, these features help accurately identify and classify reflective DDoS attacks.

### 4.3. Comparative Analysis of ML Models

Based on the dataset with 68 features post-preprocessing, this paper assesses the efficacy of seven different models. The specific results are shown in Table 6. NB performed the worst, with a long prediction time and low accuracy. DT and LR had the shortest prediction times, 0.2638 s and 0.2432 s, respectively, but LR’s accuracy was only 76.97%, so it was not suitable as the main prediction model. DT, RF, and KNN had similar accuracies, all around 94.5%, with KNN taking a longer prediction time of 90.0665 s. LightGBM and XGBoost performed best, with accuracies of 98.39% and 97.95%, respectively, and the difference in prediction time between the two was only 0.135 s. Therefore, this paper selected LightGBM as the attack detection model.

**Figure 5:**
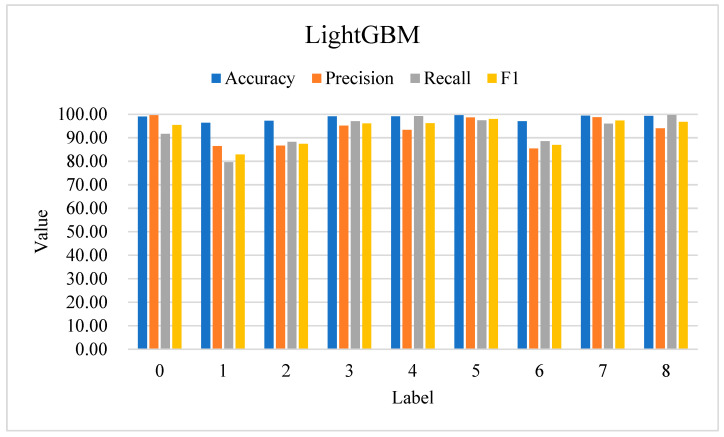
LightGBM’s performance in classifying DDoS attacks (using SMOTE).

Figure 5 presents the detection outcomes for eight reflective DDoS attacks using LightGBM. The model performs exceptionally well on attack types 0, 3, 4, 5, 7, and 8, and the four evaluation indexes all exceed 90%. However, performance for attack types corresponding to labels 1, 2, and 6 (DrDoS_DNS, DrDoS_LDAP, and DrDoS_SNMP) has shown a decline. The main reasons for this are as follows:

1. Feature Similarity Between Normal and Malicious Traffic:

These reflection attacks generate traffic that closely resembles legitimate traffic, as they exploit standard protocol requests (e.g., DNS, LDAP, SNMP). The similarity between normal and attack traffic complicates detection through conventional traffic or statistical patterns.

2. Protocol Complexity and Redundant Responses:

Protocols like DNS, LDAP, and SNMP inherently allow large response sizes and contain vulnerabilities (e.g., DNS amplification via EDNS). The high redundancy in responses makes distinguishing normal traffic from malicious traffic particularly challenging, especially during high-load conditions.

3. Dynamic Traffic Patterns:

DrDoS attacks often employ distributed reflection mechanisms, dispersing the attack across multiple reflectors, resulting in minor changes in traffic patterns per reflector. Attackers may also randomize their traffic patterns, making it difficult to detect anomalies using traditional methods.

4. Invisibility of the Victim:

The indirect nature of reflection attacks means the true victim is not easily visible in the traffic logs of the reflectors, which limits the effectiveness of Source IP-based detection techniques.

Specifically, the classification findings of label 1 (DrDoS_DNS) require further optimization to enhance the model’s prediction accuracy and recall. The performance of labels 2 (DrDoS_LDAP) and 6 (DrDoS_SNMP) also shows potential for improvement, especially in terms of improving precision. These three attack types usually have complex and varied feature patterns. For example, a DrDoS_DNS attack may appear as a large number of DNS requests, but these requests may be similar to normal DNS traffic patterns and difficult to effectively distinguish by simple feature extraction methods. In the next section, we will use GWO to optimize the hyperparameters of LightGBM to further optimize the performance of attack types corresponding to labels 1, 2, and 6 (DrDoS_DNS, DrDoS_LDAP, and DrDoS_SNMP).

**Figure 6:**
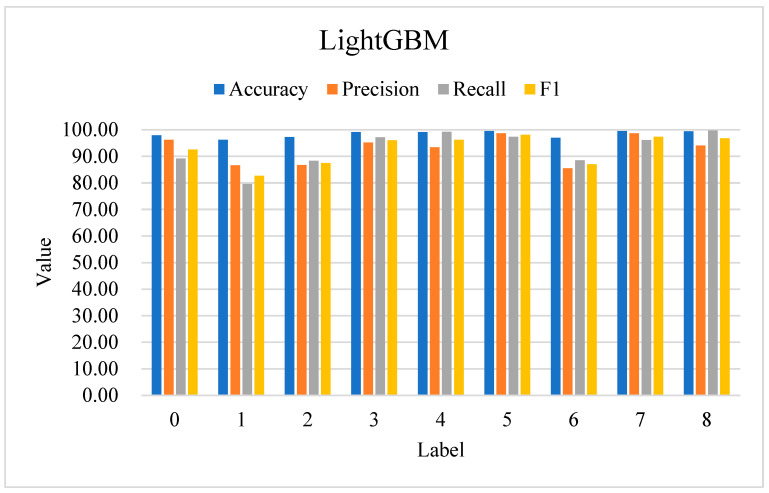
LightGBM’s performance in classifying DDoS attacks (SMOTE is not used).

As shown in Figure 6, based on the experimental results without synthetic augmentation, the model demonstrates a notable impact on performance, particularly for the minority class (label 0). When SMOTE was used, label 0 achieved a recall of 91.62% and an F1-score of 95.42%. Without SMOTE, the recall and F1-score for label 0 decreased to 89.15% and 92.58%, respectively, indicating that the imbalance in the dataset led to a reduced ability to detect normal traffic. However, in real-world scenarios, normal traffic typically constitutes a much larger portion of the network traffic compared to attack traffic, which means this performance decline may not significantly affect practical application. For labels 1–8, which already had balanced and sufficient samples, minimal changes in performance were observed. This suggests that while synthetic augmentation improves the detection of underrepresented classes, the model remains robust for the majority of classes even when trained on imbalanced data. Therefore, it is expected to generalize well in realistic network environments, where normal traffic predominates.

Table 7 shows the evaluation indicators of the eight features selected by the four ML methods and the eight features selected by the BPSO-SA method proposed in this paper for the seven ML models. The results indicate that combining the BPSO-SA FS method with the LightGBM classification model yields the highest performance, with accuracy at 98.32%, precision at 98.38%, recall at 98.36%, and an F1-score of 98.32%, while achieving a prediction time of just 0.2399 s. The RF FS method combined with the LightGBM and XGBoost models also performs well, with accuracies of 97.32% and 96.92%, F1-scores of 97.32% and 96.91%, and low prediction time. In addition, the KNN model has high accuracy, but its prediction time is much higher than other models. The DT and RF models perform slightly worse, while NB performs the worst, with an accuracy of no more than 52.01%. In summary, the BPSO-SA FS method combined with the LightGBM model provides the best classification performance and low prediction time.

**Figure 7:**
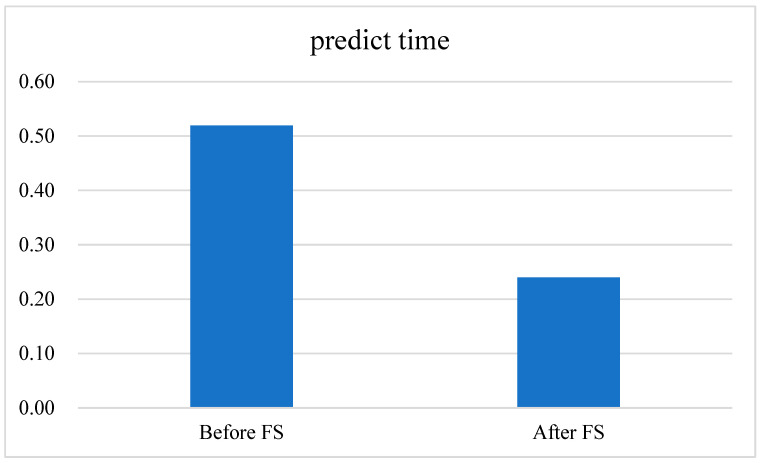
Comparison of prediction time before and after FS.

After BPSO-SA FS, not only is the number of features used in the end determined but these features are also specifically listed, such as Bwd IAT Std, Flow Byts/s, etc. The number of features decreased from 68 to 8, representing an 88.23% reduction. Additionally, the prediction time is cut from 0.5195 to 0.2399 s, reflecting a 53.82% decrease, as illustrated in Figure 7.

**Figure 8:**
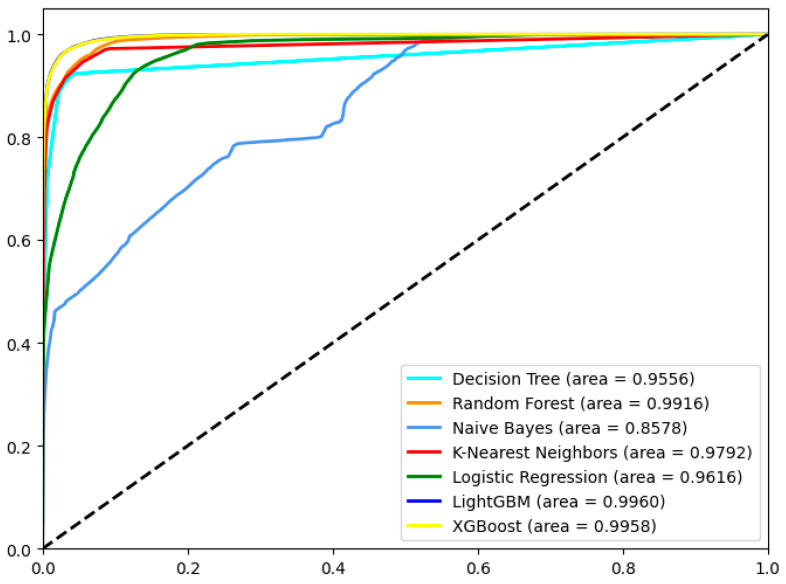
ROC curves of 7 ML models after FS using the BPSO-SA method.

**Figure 9:**
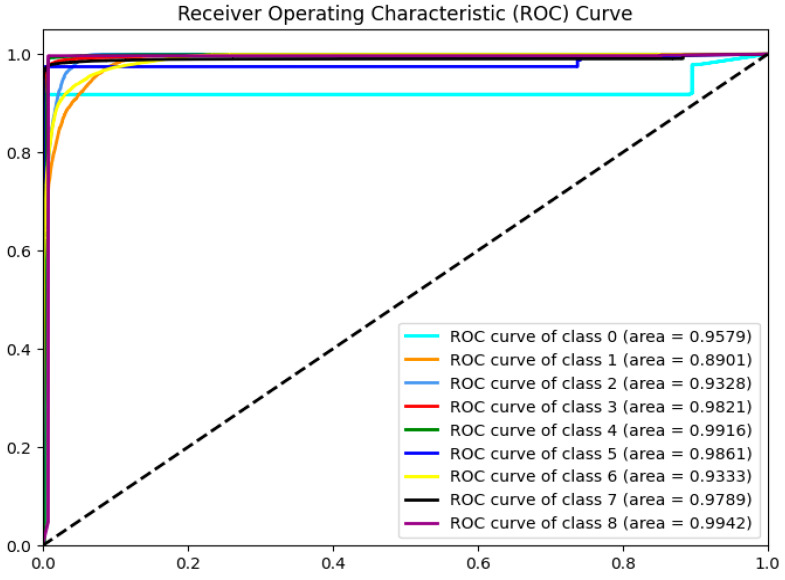
Classification results of 8 types of reflection DDoS attacks using LightGBM.

Figure 8 and Figure 9 show the ROC curves of seven machine learning models after FS using the BPSO-SA method, and the results of classifying eight reflective DDoS attacks using LightGBM. As seen in Figure 8, the LightGBM model performs best, with an area under the ROC curve of 0.9960. The second is the XGBoost model, with an area under the ROC curve of 0.9958. The NB model performs the worst, with an AUC of only 0.8578.

As can be observed from Figure 9, the classification results of the LightGBM model for labels 1, 2, and 6 are slightly inferior to those of other labels.

### 4.4. GWO Optimizes LightGBM Hyperparameters

To enhance the LightGBM model’s classification accuracy for labels such as DrDoS_DNS, DrDoS_LDAP, and DrDoS_SNMP, we utilized the GWO algorithm to optimize the hyperparameters of LightGBM. GWO’s unique strengths lie in its effective balance between exploration and exploitation, enabling it to navigate complex search spaces and avoid local optima.

In our implementation, we configured the GWO with a wolf pack size of 20 and set the number of iterations to 20, allowing for an exhaustive search of the hyperparameter space. Key hyperparameters optimized by GWO included the learning rate, the maximum depth of the trees, the number of leaves, and the regularization parameters (L1).

We chose GWO over other swarm intelligence algorithms because of its superior convergence behavior and robustness in handling high-dimensional problems. GWO mimics the leadership hierarchy and hunting mechanisms of grey wolves, which allows it to effectively balance the exploration of the search space with the exploitation of known good solutions. This unique approach reduces the risk of getting trapped in local optima compared to other methods, which can be more susceptible to premature convergence.

**Figure 10:**
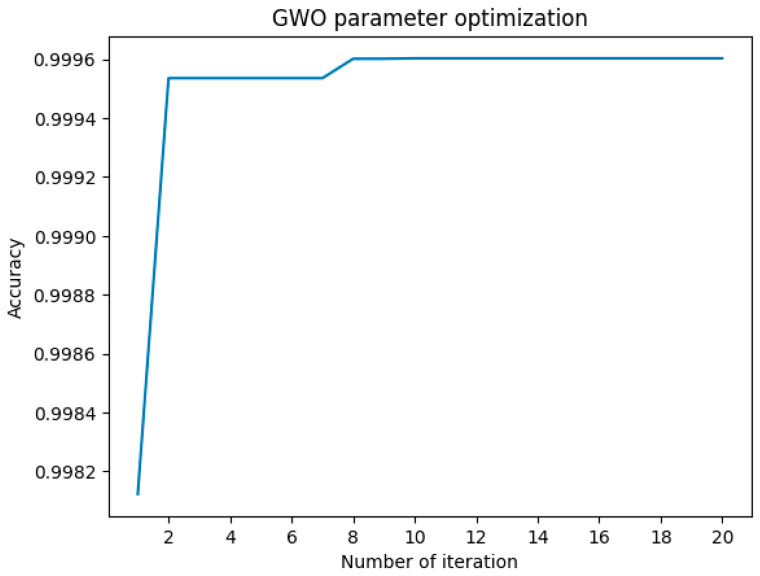
Accuracy change in LightGBM hyperparameters optimized by GWO algorithm.

The results, depicted in Figure 10, illustrate a significant improvement in accuracy throughout the iterations. This optimization led to a more robust model capable of enhancing detection performance, specifically for the identified attack types. The optimized LightGBM model demonstrates enhanced sensitivity to the characteristics of DrDoS_DNS, DrDoS_LDAP, and DrDoS_SNMP attacks, which often involve complex and subtle variations in network traffic.

The improvements in detection accuracy for these DDoS attacks are attributed to GWO’s ability to refine the model’s parameters effectively. This leads to better feature utilization, as the model can leverage the most informative features for classification. Enhanced classification performance results from GWO’s capacity to find optimal hyperparameter configurations that adapt to the unique data distributions associated with these attack types. The optimal hyperparameter configuration obtained through GWO is summarized in Table 8, highlighting the adjustments made to further improve the detection accuracy for these DDoS attacks.

The selection of these hyperparameters was based on their substantial influence on both the performance of the model and the training procedure. The num_leaves and max_depth parameters control the complexity of the model. The min_data_in_leaf, bagging_fraction, feature_fraction, and lambda_l1 parameters help prevent overfitting. Meanwhile, the learning_rate has an impact on both the speed at which the model converges and its stability. By tuning these key parameters, both the training efficiency and final performance can be improved, enhancing the model’s generalization ability.

Compared with the default settings of LightGBM, the optimized hyperparameters are adjusted by modifying the number of leaf nodes, increasing the minimum number of samples per leaf node, reducing the sampling ratio of data and features, limiting the maximum depth of the tree, slightly reducing the learning rate, and introducing L1 regularization. These adjustments significantly improve the classification performance of the model. Regarding the evaluation indicators, namely accuracy, precision, recall, and F1-score, all of them exhibit superior performance. At the same time, these optimizations also significantly shorten the prediction time and improve the calculation efficiency, making the model more efficient and reliable in practical applications.

**Figure 11:**
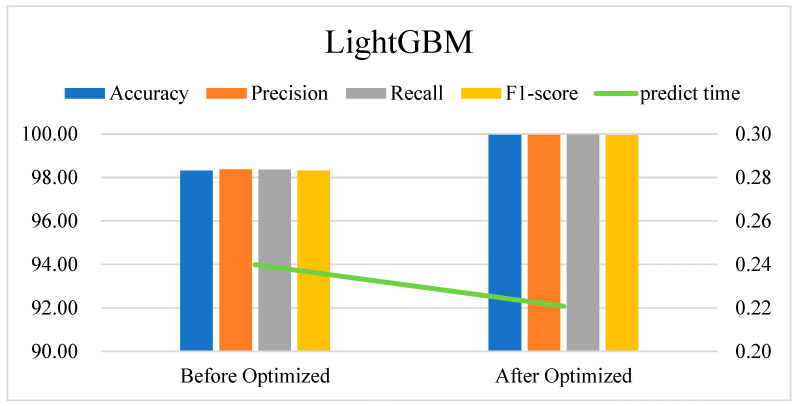
Comparison of LightGBM model performance in DDoS attack classification pre- and post-optimization.

The results of the DDoS attack classification using the optimized LightGBM model are shown in Figure 11. The optimized model has significantly improved in various evaluation indicators: the accuracy has improved by 1.64%, precision has risen by 1.58%, recall has increased by 1.61%, and the F1-score has gone up by 1.63%. Additionally, the prediction time is reduced by 0.0191 s. These enhancements not only increase the model’s classification performance but also make it more efficient in actual applications.

### 4.5. Comparison with Previous Studies

Table 9 shows the comparison results of our method with existing research. Compared with other methods, BPSO-SA-GWOLightGBM performs significantly better on the CICDDoS2019 dataset. The method achieves an accuracy of 99.96% while performing well in terms of precision (99.96%), recall (99.97%), and F1-score (99.95%), significantly surpassing most existing methods such as Transformer (98.58% accuracy) and DNN (94.57% accuracy). In addition, BPSO-SA-GWOLightGBM uses only eight features but still provides excellent performance, with a prediction time of only 0.2208 s, demonstrating efficient computing power and excellent FS effects. These results show that BPSO-SA-GWOLightGBM has significant advantages in terms of classification performance, computational efficiency, and FS.

## 5. Conclusions

This work offers a novel FS approach that combines BPSO with SA and uses GWO to optimize the hyperparameters of the LightGBM model, resulting in a new reflection-based DDoS attack detection model. The experimental results demonstrate that this method performs exceptionally well on the CICDDoS2019 dataset, significantly out-performing existing intrusion detection techniques.

However, while our model excels in detecting reflection-based DDoS attacks, its effectiveness in addressing other types of DDoS attacks remains untested. Extending the model to encompass a broader range of attack types could enhance its generalization and robustness against diverse network threats. Additionally, the model has yet to be evaluated in real-world environments with dynamic network conditions, where its adaptability to evolving attack characteristics and traffic patterns is crucial. Furthermore, the BPSO-SA algorithm, combined with GWO for hyperparameter optimization, can be computationally expensive, particularly with large-scale, high-dimensional datasets, leading to increased training times due to the multiple iterations required for convergence.

Future research should prioritize improving the diversity of FS approaches, expanding the model to encompass other forms of network attacks, and strengthening real-time detection capabilities. Additionally, the performance of the BPSO-SA and GWO algorithms is highly sensitive to hyperparameter settings, and different parameter combinations may lead to fluctuations in model performance. Further tuning and experiments are needed to identify the optimal configuration. By optimizing and refining the algorithm, the overall performance and applicability of the model can be enhanced, thereby advancing network security detection technology and better addressing complex and diverse network attack scenarios. Furthermore, exploring the model’s scalability and adaptability in dynamic, real-world environments will be invaluable. Future work should focus on enhancing the model’s ability to adapt to evolving network attack characteristics and traffic patterns, improving its reliability and practical utility in real-world applications.

## Figures and Tables

**Table 1:** Comparison of the literature.

Ref.	Method	Advantages	Limitations
Nguyen et al. [16]	GMM BILSTM	Can correctly and elegantly handle new traffic and old traffic.	When encountering new attacks, the system performance will be seriously degraded.
Javeed et al. [17]	CuDNNLSTM CuDNNGRU	This technology is scalable and reasonably priced; it can identify threats in an Internet of Things (IoT) context.	To increase the model’s capacity for generalization, more investigation is required.
Aldhyani et al. [6]	CNN-LSTM	Detection of DDoS attacks in agricultural 4.0 environment.	Limited by standard datasets.
Arden et al. [22]	DT, NB, RF, LightGBM, Catboost, XGBoost	Random forest can perform best in some cases without hyperparametric tuning.	Does not consider all types of datasets and algorithms.
Hsu et al. [23]	RF, GBDT, XGBoost, LightGBM, CatBoost, KNN, MLP	The proposed method is applicable in real time to large-scale networks.	The study of real data may require more resources and time to collect and process data.
Altalbe et al. [24]	FFS-IDS, DT, RF	It can effectively detect DDoS, gear spoofing, and RPM spoofing attacks.	The ensemble learning method may require more computing resources and tuning.
Hasan et al. [25]	LR, SVM, DT, RF, ANN	It provides an intelligent, safe, and reliable infrastructure based on IoT, which can detect its vulnerabilities.	When the algorithm is actually deployed, it could be necessary to take into account its real-time performance and flexibility in response to novel attack types.
Alzahrani et al. [26]	KNN, SVM, NB, DT, RF, LR	It can effectively identify and defend DDoS attacks in the IoT environment.	There is no mention of real-time performance.

**Table 2:** Feature numbers and feature names.

No.	Feature Name	No.	Feature Name	No.	Feature Name
1	ACK Flag Cnt	24	Flow IAT Min	47	Init Bwd Win Byts
2	Active Max	25	Flow IAT Std	48	Init Fwd Win Byts
3	Active Mean	26	Flow Pkts/s	49	Label
4	Active Min	27	Fwd Act Data Pkts	50	Pkt Len Max
5	Active Std	28	Fwd Header Len	51	Pkt Len Mean
6	Bwd Header Len	29	Fwd IAT Max	52	Pkt Len Min
7	Bwd IAT Max	30	Fwd IAT Mean	53	Pkt Len Std
8	Bwd IAT Mean	31	Fwd IAT Min	54	Pkt Len Var
9	Bwd IAT Min	32	Fwd IAT Std	55	Pkt Size Avg
10	Bwd IAT Std	33	Fwd IAT Tot	56	Protocol
11	Bwd IAT Tot	34	Fwd Pkt Len Max	57	RST Flag Cnt
12	Bwd Pkt Len Max	35	Fwd Pkt Len Mean	58	Subflow Bwd Byts
13	Bwd Pkt Len Mean	36	Fwd Pkt Len Min	59	Subflow Bwd Pkts
14	Bwd Pkt Len Min	37	Fwd Pkt Len Std	60	Subflow Fwd Byts
15	Bwd Pkt Len Std	38	Fwd Pkts/s	61	Subflow Fwd Pkts
16	Bwd Pkts/s	39	Fwd PSH Flags	62	SYN Flag Cnt
17	Bwd Seg Size Avg	40	Fwd Seg Size Avg	63	Tot Bwd Pkts
18	CWE Flag Count	41	Fwd Seg Size Min	64	Tot Fwd Pkts
19	Down/Up Ratio	42	Idle Max	65	TotLen Bwd Pkts
20	Flow Byts/s	43	Idle Mean	66	TotLen Fwd Pkts
21	Flow Duration	44	Idle Min	67	Unnamed: 0
22	Flow IAT Max	45	Idle Std	68	URG Flag Cnt
23	Flow IAT Mean	46	Inbound		

**Table 3:** Label serial number and sample number corresponding to reflective DDoS attacks.

Attack Type	Subset No.	Number
BENIGN	0	100,000
DrDoS_DNS	1	100,000
DrDoS_LDAP	2	100,000
DrDoS_MSSQL	3	100,000
DrDoS_NetBIOS	4	100,000
DrDoS_NTP	5	100,000
DrDoS_SNMP	6	100,000
DrDoS_SSDP	7	100,000
TFTP	8	100,000

**Table 4:** BPSO-SA algorithm initial parameter settings.

Parameter	Value
min_selected_features	2
num_particles	50
num_iterations	500
ω	0.4
$c_{1}$	2
$c_{2}$	2
$T_{max}$	1
$T_{min}$	0.0001

**Table 5:** Features selected by different FS methods.

FS Method	Sub-Features Selected
XGBoost	27, 34, 37, 50, 52, 55, 64, 67
RF	34, 35, 36, 40, 50, 52, 55, 67
DT	1, 20, 21, 46, 48, 52, 67, 68
IG	35, 36, 40, 50, 51, 52, 55, 67
BPSO-SA	10, 20, 21, 22, 35, 41, 54, 67

**Table 6:** Evaluation indicators of 7 ML models on all features.

Model	Accuracy	Precision	Recall	F1-Score	Predict Time
DT	94.53	94.59	94.51	94.55	0.2638
RF	94.44	94.71	94.47	94.02	8.4510
NB	52.99	73.04	52.80	52.15	2.7771
KNN	94.60	94.68	94.60	94.62	90.0665
LR	76.97	79.11	76.95	77.14	0.2432
LightGBM	98.39	98.36	98.45	98.34	0.5195
XGBoost	97.95	97.93	97.90	97.91	0.6545

**Table 7:** Performance of different FS methods on different ML models.

FS Method	Model	Accuracy	Precision	Recall	F1-Score	Predict Time
XGBoost	DT	93.01	93.06	93.00	92.99	0.2465
	RF	92.83	93.07	92.78	92.34	1.2874
	NB	50.98	70.89	50.66	50.10	1.1256
	KNN	93.10	93.17	93.12	93.10	81.2511
	LR	75.00	77.19	75.03	75.20	1.9751
	LightGBM	97.00	97.06	97.04	97.00	0.2340
	XGBoost	96.50	96.49	96.50	96.49	0.3466
RF	DT	92.51	92.56	92.50	92.50	0.2471
	RF	92.41	92.62	92.49	92.03	1.2770
	NB	50.76	71.89	51.09	51.15	1.2024
	KNN	92.60	92.67	92.69	92.69	81.2654
	LR	75.96	78.25	75.99	76.15	1.0031
	LightGBM	97.32	97.38	97.36	97.32	0.2576
	XGBoost	96.92	96.91	96.92	96.91	0.3479
DT	DT	92.00	92.05	92.00	91.97	0.2443
	RF	91.87	92.05	91.63	91.32	1.2478
	NB	50.51	71.34	50.66	50.64	1.1078
	KNN	92.10	92.17	92.12	92.10	81.2903
	LR	75.50	77.19	75.52	75.70	1.1042
	LightGBM	96.50	96.56	96.54	96.50	0.2354
	XGBoost	95.98	95.90	95.92	95.99	0.3340
IG	DT	91.51	91.56	91.50	91.55	0.2671
	RF	91.41	91.66	91.42	91.07	1.2566
	NB	51.07	71.88	51.47	51.82	1.2428
	KNN	91.60	91.67	91.68	91.60	81.2819
	LR	75.96	78.11	75.87	76.15	1.1297
	LightGBM	96.38	96.38	96.36	96.89	0.3614
	XGBoost	95.92	95.92	96.89	96.98	0.3601
BPSO-SA	DT	94.51	94.56	94.50	94.50	0.2470
	RF	94.40	94.67	94.48	94.15	1.2570
	NB	52.01	72.86	52.62	52.10	1.1128
	KNN	94.60	94.67	94.03	94.60	81.1511
	LR	76.96	79.24	76.91	77.15	0.9892
	LightGBM	98.32	98.38	98.36	98.32	0.2399
	XGBoost	97.92	97.93	97.92	97.91	0.3354

**Table 8:** Optimal hyperparameter configuration of LightGBM model after GWO algorithm optimization.

Hyperparameter	Default	Optimized
num_leaves	31	24
min_data_in_leaf	20	22
bagging_fraction	1.0	0.33122457815348816
feature_fraction	1.0	0.5835200814259994
max_depth	−1	7
learning_rate	0.1	0.08938544625237098
lambda_l1	0.0	0.0113982727421401

**Table 9:** Research on comparative existence.

Literature	Dtaset	Method	FS Number	Class	Accuracy	Precision	Recall	F1-Score	Predict Time
[7]	CICDDoS2019	EDRFS	24	2	99.99	99.99	99.99	99.99	0.4
[10]	CICDDoS2019	CNN + BiLSTM	24	2	94.52	94.74	92.04	93.44	
[12]	NSL-KDD	RF-PSO	10	5	99.32	99.37		99.31	
[13]	NSL-KDD	ACO-SVM	8	3	99.90	99.63			0.85
[40]	CICDDoS2019	Transformer	86	11	98.58	98.82	98.66	98.48	
[41]	CICIDS2017	LASSO + LightGBM	86	2	99.77				
[42]	CICDDoS2019	BaysFusCNN	42	14	99.79	98.57	98.55	98.56	
[43]	CICDDoS2019	DNN	69	14	94.57	80.49	95.15	87.21	
our	CICDDoS2019	BPSO-SAGWOLightGBM	8	9	99.96	99.96	99.97	99.95	0.2208

## Data Availability

The CICDDoS2019 dataset is available at https://www.unb.ca/cic/datasets/ddos-2019.html (accessed on 25 December 2023).
